# Supplementary material for: The Gut Bacteria Dysbiosis Contributes to Chronic Graft-Versus-Host Disease Associated With a Treg/Th1 Ratio Imbalance
Source: Front Microbiol. 2022 Sep 8;13:813576. doi: 10.3389/fmicb.2022.813576 (PMC9493085; doi:10.3389/fmicb.2022.813576)

_MD3: 0.954708659636 (of 0.999999999999) from 19 categories displayed, excluding 0.0452913403636 from 90 categories ('All Other Categories')

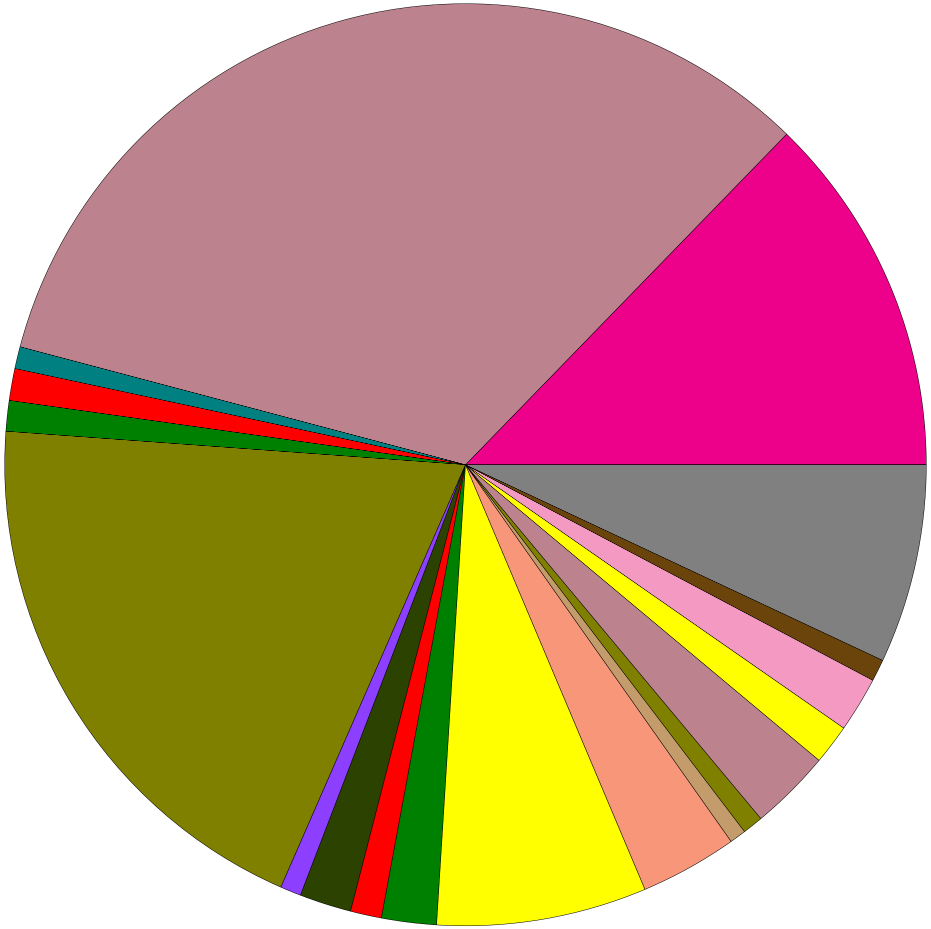

Supplement: Supplementary file 1 [file Data_Sheet_1.zip › P101SC18090073-01-B1-3-4_result/05.WebShow/taxa_charts_html/charts/xaPgErlYohUIU4MOc7zcResQ0wF55C.pdf]
